# Heterogeneity-based stratification identifies CKMT2 as a prognostic marker in osteosarcoma

**DOI:** 10.3389/fcell.2026.1822741

**Published:** 2026-05-25

**Authors:** Xuesong Li, Guanghao Li, Tianchou Peng, Junjun Qiu, Shengwei Li, Jingfu Wang, Jin Zhang

**Affiliations:** 1 Department of Pediatric Oncology, Shandong Cancer Hospital and Institute, Jinan, Shandong, China; 2 Department of Urology, Shandong Cancer Hospital and Institute, Jinan, China; 3 Department of Spine, East Hospital, Tongji University School of Medicine, Shanghai, China; 4 Department of Orthopedic Surgery, Institute of Digital Medicine, Nanjing First Hospital, Nanjing Medical University, Nanjing, China; 5 Department of Interventional Radiology, Shandong Cancer Hospital and Institute, Jinan, Shandong, China; 6 Department of Orthopedics, Nanjing First Hospital, Nanjing Medical University, Nanjing, Jiangsu, China

**Keywords:** biomarker, CKMT2, intratumoral heterogeneity, molecular dynamics simulation, osteosarcoma, prognosis

## Abstract

**Background:**

Osteosarcoma (OS) is the most common primary malignant bone tumor. Intratumoral heterogeneity (ITH) contributes to therapy resistance and disease spread. Understanding the molecular structure and cellular groups contributing to ITH, as well as their clinical impacts, remains limited.

**Methods:**

To build our investigation, we started by integrating single-cell transcriptomic data with multi-cohort transcriptomic datasets sourced from public repositories. Our bioinformatics pipeline was then designed to systematically unpack this information: first, we identified malignant cells by inferring their copy number variation (CNV); next, we assessed the degree of heterogeneity and defined distinct molecular subtypes; and finally, we conducted an in-depth analysis of the functional pathways mediating these heterogeneous characteristics. To sharpen the prognostic model linked to ITH, we employed machine learning to develop and validate a prognostic risk model for ten critical genes and performed both clinical and functional assays. Meanwhile, we validated the pro-tumorigenic role and therapeutic viability of CKMT2 through immunohistochemistry (IHC) and a series of *in vitro* functional assays.

**Results:**

Analysis of transcriptomic data from single-cells highlighted ten primary cell types in osteosarcoma. The dominant malignant population as per CNV analysis has been identified as osteoblastic cells (OBs). The subsequent subclassification of OB led to the discovery of five malignant subtypes, resulting in stratification of high and low ITH. Consensus clustering, driven by the differential expression genes discovered in ITH, successfully divided the population into two distinct molecular subtypes. These subtypes exhibited a robust correlation with clinical prognosis and immune infiltration in multiple independent cohorts. Through machine learning modeling, we identified a highly promising therapeutic target, Creatine Kinase Mitochondrial 2 (CKMT2), and performed both clinical and functional assays. IHC validated high CKMT2 expression in tumor tissues corresponding to clinical stage, and *in vitro* CKMT2 knockdown significantly decreased OS cell proliferation and colony formation.

**Conclusion:**

This study provides a systematic map of osteosarcoma’s ITH landscape, identifying distinct molecular subtypes with varying clinical outcomes and immune profiles. CKMT2 is established as a key driver of osteosarcoma progression, serving both as a prognostic biomarker and a potential therapeutic target.

## Introduction

For decades, the clinical narrative of Osteosarcoma (OS) has been defined by a stubborn reality: despite our best multimodal efforts, patient outcomes—especially for those with recurrent or metastatic disease—remain trapped at a 20%–30% survival ceiling (Gaspar et al., 2018; Bacci and Lari, 2001; Bishop et al., 2016; Meyers et al., 2005; Ritter and Bielack, 2010). This stagnation is not for lack of trying. Rather, it reflects a profound clinical impasse driven by the tumor’s elusive chemoresistance and a molecular landscape that we have yet to fully decode (Garcia-Ortega et al., 2022; Wang et al., 2025).

At the center of this failure is the tumor’s own internal diversity. OS is far from a uniform mass; it is a shifting mosaic of cellular subsets, or intratumoral heterogeneity (ITH), that dictates how a patient will ultimately fare (Marusyk et al., 2012). While we used to rely on bulk sequencing, that approach effectively “blinded” us to the rare, aggressive cells by averaging their signals into oblivion. Single-cell RNA sequencing (scRNA-seq) has changed the game, allowing us to finally zoom in on these individual cellular states (Wu F. et al., 2021; Levitin et al., 2018). We’ve seen the maps of this landscape before (Zhou et al., 2020; Ayers et al., 2021). But seeing the diversity is not the same as measuring its clinical impact. That is where the current gap lies. We have the technology to see the cells, but we lack a reliable way to translate that “noise” into a prognostic signal. Our study takes a different tack. By merging single-cell resolution with massive multi-cohort datasets, we did not just want to map the cells—we wanted to quantify the chaos of ITH itself. Our goal was to find the specific transcriptional drivers, like CKMT2, that actually move the needle on patient survival, offering a more granular roadmap for risk stratification than what is currently available.

## Materials and methods

### Data preprocessing

In our study, we obtained the gene expression and corresponding clinical data from the TARGET dataset (constructed by the National Cancer Institute). We also developed two independent datasets (GSE21257 and GSE39058) based on the Gene Expression Omnibus (GEO) database. The validation cohort in our study was derived from the two GSE datasets and the batch-effect correction was performed using the “sva” R package.

### Single-cell clustering and cell annotation analysis

We acquired the scRNA-seq dataset GSE162454 from GEO, comprising 6 OS samples. Our study performed the data processing and quality control using the Seurat R package (v5.1.0) (Hao et al., 2021). Low-quality cells were filtered according to the following criteria: (1) mitochondrial gene content >20%; (2) fewer than 250 detected genes; and (3) UMI counts <500 or log10GenesPerUMI <0.8. After the log-normalization, highly variable features were extracted via the “vst” method. Dimensionality reduction was achieved through Principal Component Analysis (PCA), resulting in the top 30 principal components for clustering. Cell clusters were determined using the FindNeighbors and FindClusters functions (resolution = 0.2) and annotated based on well-established marker genes in CellMarker 2.0 and prior literature (Becker et al., 2022; Wang et al., 2022; Devlin et al., 2021; Khaliq et al., 2022; Fawkner-Corbett et al., 2021; Che et al., 2021).

### Identification of malignant cells

We distinguished the malignant cells from non-malignant stromal and immune cells by inferring large-scale chromosomal copy number variations (CNVs) with the copyKAT R package (v1.0.8). After that, cells were classified as “aneuploid” (malignant) or “diploid” (non-malignant) based on a Bayesian mixture model and hierarchical clustering of gene expression (Gao et al., 2021).

### Analysis of stemness and trajectory

Our study assessed the differentiation potential and cellular stemness of malignant osteoblasts (OBs) using CytoTRACE (v0.3.3) (Gulati et al., 2020), with higher scores indicating a more stem-like, less differentiated state. Pseudotime trajectory analysis was conducted using Monocle 2 (v2.24.1) (Trapnell et al., 2014) to reconstruct the developmental lineage of malignant subpopulations. Dimensionality reduction was performed using the “DDRTree” algorithm, with the trajectory root defined as the cluster exhibiting the lowest differentiation score.

### Quantification and grouping of intratumoral heterogeneity (ITH)

We computed the Heterogeneity scores to quantify ITH levels in each sample, mainly presenting the distribution of malignant cells in the principal component domain, as previously reported (Ma et al., 2019). The ITH index was calculated from the average Euclidean distance of malignant cells from the centroid of the sample. According to the median of the ITH index, all the samples were stratified into high-ITH (ITHhi, n = 3) and low-ITH (ITHlo, n = 3) groups.

### Single-cell functional and interaction analysis

In our study, downstream bioinformatics analyses were conducted to characterize the functional states of malignant subpopulations comprehensively. Firstly, metabolic heterogeneity was quantitatively evaluated using the scMetabolism R package (v0.2.1) (Wu et al., 2022), which can verify the activity of metabolic pathways based on KEGG and Reactome databases using the ssGSEA algorithm. After that, the SCENIC (Single-Cell rEgulatory Network Inference and Clustering) workflow (Aibar et al., 2017) was utilized to reconstruct the gene regulatory network (GRN) and master regulators. Using weighted gene correlation network analysis, co-expression modules were generated. Meanwhile, the regulon activity scores (RAS) were computed for each cell population, allowing the transcription factor (TF) activity comparisons between the ITH-high and ITH-low groups. Ultimately, we inferred the intercellular communication networks via the CellChat R package (v1.6.0) (Jin et al., 2021). The communication probabilities were computed based on the expression of ligand-receptor pairs derived from the CellChatDB dataset, and the signaling pathways that crucially mediate the interactions between malignant cells and the TME were determined.

### Functional enrichment and pathway analysis

Our study performed the functional enrichment analyses, including Gene Ontology (GO) (Wu T. et al., 2021) and Kyoto Encyclopedia of Genes and Genomes (KEGG) (Kanehisa et al., 2021), using the clusterProfiler R package to assess the functions and signaling pathways related to differential expression genes (DEGs). Statistical significance referred to an adjusted *p*-value <0.05. Additionally, Gene Set Enrichment Analysis (GSEA) (Subramanian et al., 2005) and Single-Sample GSEA (ssGSEA) were conducted to quantify pathway activity scores for individual cells or samples using the HALLMARK gene sets (Liberzon et al., 2015).

### Differential expression gene (DEGs) analysis and consensus clustering

DEGs between the ITHhi and ITHlo groups were analyzed using the FindMarkers function in Seurat (Wilcoxon rank-sum test) with thresholds of |log_2_(FoldChange)| > 1 and an adjusted p-value <0.05. Based on the DEG analysis, unsupervised consensus clustering was performed via the ConsensusClusterPlus software package to establish robust molecular subtypes, whose prognostic differences were assessed via Kaplan-Meier (KM) survival analysis.

### Construction and validation of the prognostic model

As reported in the previous studies, a robust and consensus prognostic signature was effectively established based on an integrative machine learning framework. A total of 20 prognosis-related candidate genes were selected from the ITH-associated DEGs in the TARGET-OS cohort through univariate Cox regression (*p* < 0.05). Subsequently,94 machine learning algorithm combinations, integrating 10 screening methods (such as LASSO, Ridge, and Elastic Net) with modeling methods, were tested and optimized. The hyperparameter tuning and primary model selection were performed using cross-validation within the TARGET training cohort. After the Stepwise Cox regression (backward direction) and CoxBoost, the validation datasets (GSE21257, GSE39058, and their combined Meta-cohort after batch-effect correction) were subsequently used to independently evaluate the prognostic performance and generalizability of the selected optimal model (Binder and Schumacher, 2008). The risk score was calculated as follows:Risk Score = ∑i = 1n (Expi × βi), where Expi denotes the expression level of gene i. Patients were stratified into high- and low-risk groups based on the risk scores. The performance of our model was evaluated using KM survival analysis and time-dependent Receiver Operating Characteristic (ROC) curves (Blanche et al., 2013) in the training and independent validation datasets.

### PPI network analysis and drug screening

We performed a protein-protein interaction (PPI) network analysis for the 10 key genes in the prognostic model to identify core regulatory genes, using the STRING (v11.0) (Szklarczyk et al., 2019) database and the NetworkAnalyst 3.0 platform. Based on the PPI network and the DrugBank (Wishart et al., 2018) database, the Network Proximity algorithm was used for drug screening. This algorithm computes the shortest path distance between drug targets and the CKMT2-associated gene sets in the PPI network, identifying potential targeted drugs with statistical significance evaluated via z-score based on random simulation correction.

### Molecular docking and molecular dynamics simulation

We screened small-molecule drugs from the DrugBank database explore potential targeted drugs for the key prognostic gene CKMT2. Molecular docking was performed using AutoDock Tools 1.5.6 and AutoGrid 4 with a Lamarckian Genetic Algorithm to assess the binding affinity of drugs to the CKMT2 protein. The target protein structure (PDB ID: 4Z9M) was preprocessed using the Protein Preparation Wizard in Schrodinger software, including bond order reassignment, geometric isomer correction, missing residue and loop completion, water molecule removal, and polar hydrogen addition. The grid box was centered on the spatial position of the original co-crystallized ADP ligand. The complex with the optimal binding energy was selected,and A 100-nanosecond molecular dynamics simulation was conducted using GROMACS 2019 software to evaluate the binding stability. The simulation employed the Amber FF03 force field for the protein, while partial charges for the ligand (Prednisolone phosphate) were derived using the RESP method after electrostatic potential calculation at the B3LYP/6-311G** level in Gaussian 09. The protein-ligand complex was placed in a 10 × 10 × 10 Å orthorhombic box, solvated with the TIP3P water model, and neutralized with 0.15 M Na+ and Cl-ions. Following 1,000 steps of energy minimization,the system was equilibrated for 100 ps under NVT and 100 ps under NPT ensembles, using the Nose-Hoover thermostat and Martyna-Tobias-Klein barostat, respectively. The 100 ns production run was performed at 300 K with a 2 fs time step, saving energy and coordinate files every 10 ps.

### Immunohistochemical analysis and functional experiment

The OS tissue microarray (L1024901) was obtained from Xi’an Zhongke GuanghuaBiotechnology Co., Ltd. (https://bioaitech.com). The TMA contained 102 tissue cores from 51patients, including 50 osteosarcoma patients (mean age 27.8 years; 31 males and 19 females)and 1 normal bone tissue control. Each patient contributed two cores. Among the OS patients,the clinical stages included Stage IIA (n = 12), IIB (n = 33), and IVB (n = 5). The IHC scoringwas performed by two independent pathologists blinded to the clinical data. To assess the CKMT2 impacts on cell proliferation and colony formation, we carried out the CCK-8 assays and plate colony formation experiments, followed by the CKMT2 knockdown using CKMT2-specific small interfering RNAs (siRNAs). Three siRNAduplexes targeting CKMT2 (homo-ID:1,160) were designed and synthesized with HPLCpurification: si-CKMT2-1 (target position 861): sense 5′-GGA​UAA​AUG​AGG​AGG​AUC​ATT-3′, antisense 5′-UGA​UCC​UCC​UCA​UUU​AUC​CTT-3'; si-CKMT2-2 (target position 1,279): sense 5′-GGA​GAG​AGG​CCA​AGA​UAU​UTT-3′, antisense5′-AAUAUCUUGGCCUCUCUCCTT-3'; si-CKMT2-3 (target position 381): sense 5′-UCA​UAA​AGA​CUG​UGG​GCA​UTT-3′, antisense 5′-AUG​CCC​ACA​GUC​UUU​AUG​ATT-3'. Anon-targeting siRNA (sense 5′-UUC​UCC​GAA​CGU​GUC​ACG​UTT-3′, antisense 5′-ACG​UGA​CAC​GUU​CGG​AGA​ATT-3′) served as the negative control. The knockdownefficiency was verified by qPCR using primers CKMT2-F:5′- CCA​AGC​GCA​GAC​TAC​CCA​G -3′ and CKMT2-R:5′- GGT​GTC​ACC​TTG​TTG​CGA​AG -3′ in the 143B and U2OS cell lines. For the CCK-8 assay, cells were implanted into 96-well plates with a density of approximately 5,000 cells per well. After 24 h culture for adhesion, cells were applied to different drug concentrations and an additional 24–72 h cell culture. 10 μL CCK-8 reagent was poured into each well and incubated for 1–4 h 450 nm absorbance was measured using a microplate reader to figure out the cell viability and the half-maximal inhibitory concentration (IC_50_). For the plate clonogenic assay, cells were plated at low density (200–1,000 cells per well) in 6-well plates and cultured under standard conditions for 2–3 weeks with regular medium changes. Once the clones formed visually, the cells were fixed with methanol and stained with crystal violet. The clones containing more than 50 cells were counted to evaluate the clonogenic efficiency involving long-term proliferation capacity. Both methods are technically suitable for rapid toxicity screening of drugs and assessment of clonal potentials.

### Statistical analysis

Statistical analysis of continuous variables between two groups or among multiple groups was performed using the Mann-Whitney U test or the Kruskal–Wallis test, respectively. Categorical variables between the two groups were analyzed using the Chi-square test. In our study, statistical analyses were all conducted using R software (version 4.0.5) and GraphPad Prism 9.5. Data are expressed as mean ± standard deviation, and each experiment was independently replicated at least three times. A *p*-value <0.05 was statistically significant in our study.

## Results

### Single-cell atlas reveals the heterogeneity, malignant transformation, and differentiation trajectories of osteoblasts in osteosarcoma

**FIGURE 1:**
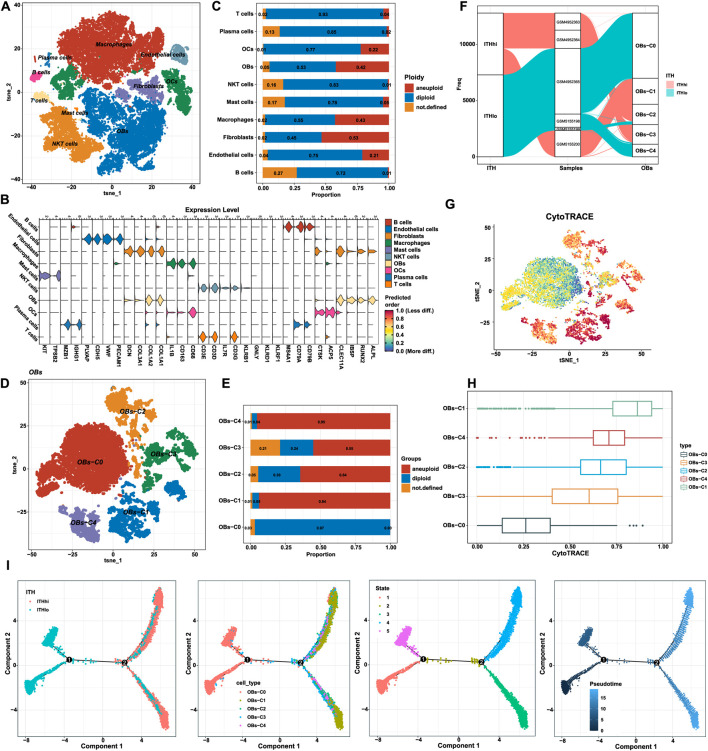
Single-cell landscape and malignant evolution of osteoblasts in osteosarcoma. **(A)** t-SNE plot illustrating the primary cell lineages found in osteosarcoma samples, with colors representing different cell types. **(B)** Violin and dot plots depicting the expression levels of standard marker genes across the identified cell clusters. Dot size represents the percentage of cells expressing the gene, and color intensity indicates the average expression level. **(C)** A bar chart showing the percentage of aneuploid (CNV-positive) and diploid (CNV-negative) cells among various cell types as determined by copyKAT. **(D)** Osteoblasts (OBs) were sub-clustered using t-SNE, resulting in five separate sub-populations, OBs-C0 to OBs-C4. **(E)** Each OB sub-cluster’s CNV status proportion is illustrated. **(F)** Sankey diagram illustrating how OB sub-clusters are distributed among samples with varying levels of intratumoral heterogeneity (ITH). **(G)** The t-SNE plot is colored according to CytoTRACE stemness scores, with red signifying greater stemness potential. **(H)** A box plot compares CytoTRACE scores across different OB sub-clusters. **(I)** The pseudotime trajectory analysis of OB sub-clusters, performed with Monocle 2, is colored according to ITH status, cell type, developmental stage, and pseudotime value.

We obtained and analyzed 6 samples from the GSE162454 dataset to comprehensively characterize the TME in OS using scRNA-seq. After the unsupervised clustering and t-SNE dimensionality reduction, there existed 10 major cell populations annotated based on the expression of specific marker genes, including OBs, plasma cells, T cells, osteoclasts (OCs), natural killer T cells (NKT), mast cells, macrophages, fibroblasts, endothelial cells, and B cells (Figures 1A,B). We utilized copyKAT to infer CNVs and classified cells as ‘aneuploid’ (withCNV alterations) or ‘diploid’ (without CNV alterations). The analysis revealed thatthe aneuploid proportion in OBs was 42%. Interestingly, fibroblasts andmacrophages, which are closely related to tumor development, also exhibitedhigh aneuploid proportions of 53% and 43%, respectively. In contrast,immune-related populations such as T cells, NKT cells,and B cells showed very lowaneuploid proportions (<5%) (Figure 1C). Given that OBs are the established cellof origin for osteosarcoma,the substantial 42% aneuploidy rate confirms theirmalignant transformation. The elevated aneuploidy signals in fibroblasts andmacrophages can be attributed to physiological processes in the bone tumormicroenvironment, such as osteoclast formation through macrophage fusion-mediated polyploidization (Takegahara et al., 2016) and tumor-stroma interactions that inducegenomic instability (Pienta et al., 2022), rather than indicating they are the primary malignantclones. In our study, the internal heterogeneity of malignant OBs was further investigated and the malignant OBs were divided into five subtypes (OBs_C0 to OBs_C4) (Figure 1D). CNV analysis revealed that there were significant genomic differences among these subtypes. The proportions of aneuploid cells in OBs_C1 and OBs_C4 were exceptionally high, at 94% and 95%, respectively, while OBs_C0 mainly consisted of diploid cells (Figure 1E). Subsequently, the samples were classified into the ITHhi and ITHlo groups according to the ITH score. A Sankey diagram visualization showed that ITHhi samples showed high proportions of OBs_C1, OBs_C2, and OBs_C3 subtypes, whereas ITHlo samples mainly involved OBs_C0 and OBs_C4 subtypes (Figure 1F), indicating a correlation between OBs subtype distribution and tumor heterogeneity. Differentiation status among OB subgroups was quantitatively analyzed using CytoTRACE scores, suggesting a significant difference in differentiation potentials (Figure 1G). Notably, the OBs_C0 subgroup showed a lower stemness score than the other subgroups, indicating a more terminally differentiated status (Figure 1H). Ultimately, we reconstructed the differentiation trajectory of OB cells using the Monocle2 algorithm. Pseudotime analysis results demonstrated that the differentiation occurs from a relatively primitive state, mainly including ITHlo or OBs_C0 cells, to a terminally differentiated state, which mainly consists of ITHhi or OBs_C1/C2/C3 cells (Figure 1I). These results systematically revealed the genomic, transcriptomic, and functional heterogeneity of OBs in the OS tumor microenvironment and different degrees of malignancy and differentiation potential in the single-cell subgroups, potentially indicating their differentiation and evolutionary relationships within the tumor ecosystem.

### Transcriptional characteristics and prognostic significance of intratumoral heterogeneity (ITH)

**FIGURE 2:**
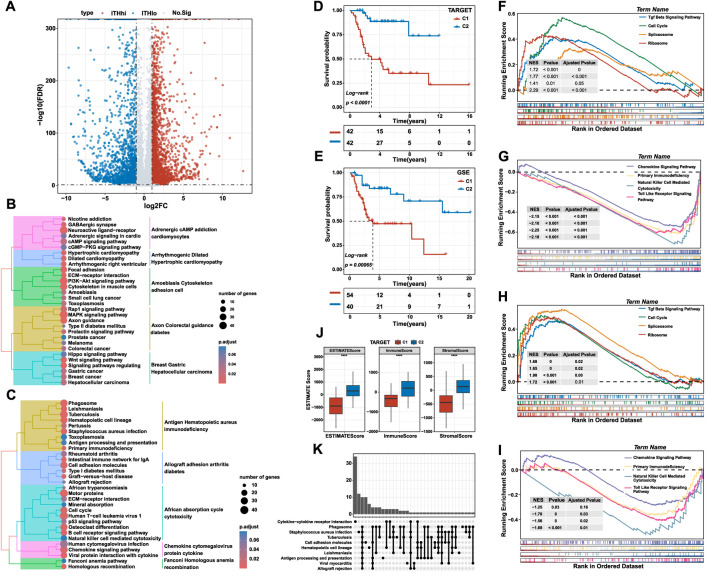
Functional characterization and prognostic significance of ITH-related genes. **(A)** The volcano plot shows the genes with differential expression between the ITHhi and ITHlo groups, with red dots indicating upregulated genes and blue dots indicating downregulated genes. **(B,C)** KEGG pathway enrichment analysis reveals the biological functions of genes upregulated in the ITHhi **(B)** and ITHlo **(C)** groups. **(D,E)** Kaplan-Meier survival analysis assesses the prognostic significance of ITH-related signatures in the TARGET **(D)** and GSE **(E)** groups, with P-values calculated using the log-rank test. **(F–I)** Gene Set Enrichment Analysis (GSEA) was used to identify enriched signaling pathways in the TARGET **(F,G)** and GSE **(H,I)** cohorts. **(J)** Box plots comparing the ESTIMATE, Immune, and Stromal scores between subtypes in the TARGET cohort. p < 0.0001. **(K)** KEGG enrichment analysis of differential expression genes between subtypes in the TARGET cohort.

In our study, we performed differential gene expression analysis in OBs to investigate the molecular mechanisms in the high and low ITH groups. The volcano plot illustrates numerous significantly DEGs between groups, indicating that the transcriptional profiles were substantially different (Figure 2A). According to the KEGG pathway enrichment analysis, the upregulated genes in the ITHhi group were significantly higher in pathways related to the tumor proliferation and signal transduction, such as the TGF-β signaling, cell cycle, PI3K-Akt, and MAPK signaling pathways (Figure 2B), whereas the upregulated genes in the ITHlo group were predominantly higher in the immune-related pathways, such as the chemokine signaling, Toll-like receptor signaling, phagosome, and antigen processing and presentation (Figure 2C). These results suggest that the two heterogeneous states correspond to different functional conditions: the ITHhi group is more likely to result in higher tumor proliferation, while the ITHlo group tends to be immune-activated. The unsupervised clustering results derived from the ITH-related DEGs in the OS tissues revealed that the C1 and C2 subtypes showed a significant difference in prognosis. In the TARGET dataset, patients with the C2 subtype achieved significantly shorter overall survival than those with the C1 subtype (Log-rank *P* < 0.0001) (Figure 2D). Similarly, the C2 subtype groups showed poorer prognosis than the C1 subgroup in the independent GSE datasets (Log-rank P = 0.00065) (Figure 2E), supporting the clinical stability and prognostic values of the C1 and C2 risk stratification. Additionally, the GSEA was performed to verify the differences in the biological functions of the C1 and C2 subtypes. Consistent with the pathway enrichment analysis in the TARGET and GSE datasets, the high-risk C2 subtype demonstrated positive enrichment in tumor proliferation-related pathways, including TGF-β signaling, cell cycle, spliceosome, and ribosomes (Figures 2F,H). Meanwhile, the C2 subtype showed significant negative enrichment in the immune-related pathways, including chemokine signaling, primary immunodeficiency, and natural killer cell-mediated cytotoxicity in both datasets (Figures 2G,I). Finally, we employed the ESTIMATE algorithm to evaluate the immune infiltration states of the TME. It was suggested that the StromalScore, ImmuneScore, and ESTIMATEScore scores in the high-risk C2 subtype were significantly lower compared to the C1 subtype (Figure 2J). Our results suggest that poor prognosis driven by high ITH is closely associated with a “cold” TME with active tumor proliferation and immune suppression. The UpSet plot highlighted the intersections of enriched pathways from various analyses, potentially underlining the subtype-specific biological processes (Figure 2K).

### Single-cell analysis of differences between tumor samples exhibiting intratumoral heterogeneity

**FIGURE 3:**
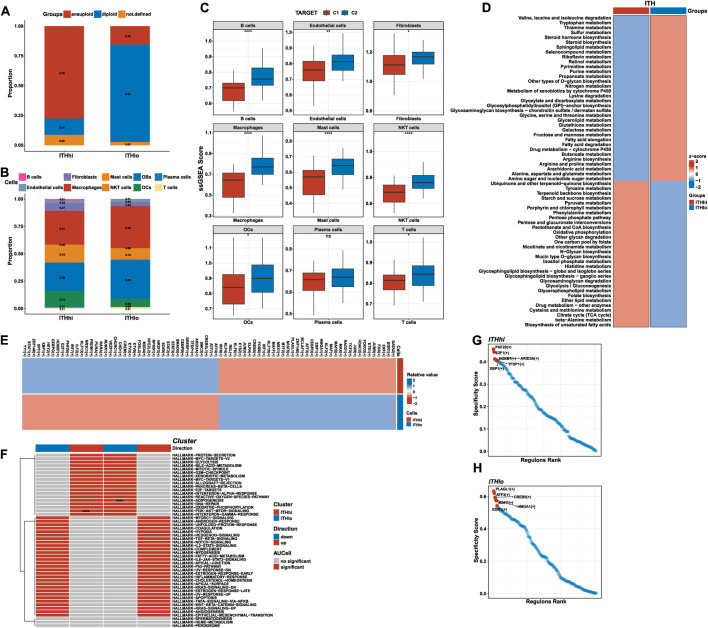
Genomic, microenvironmental, and regulatory landscapes of ITH subtypes. **(A,B)** Stacked bar graphs showing the distribution of CNV status **(A)** and cell types **(B)** in ITHhi and ITHlo categories. **(C)** Box plots illustrate the infiltration levels of immune and stromal cells in the TARGET cohort, with p-values determined by the Student’s t-test (*p < 0.05, **p < 0.01, ***p < 0.001, ****p < 0.0001). **(D)** Heatmap displaying the enrichment scores of metabolic pathways. **(E)** Heatmap showing the activity of differentially expressed regulons. **(F)** GSVA enrichment analysis of Hallmark gene sets differentiating between ITHhi and ITHlo groups. **(G,H)** Rank-ordered specificity scores pinpointing master regulators in ITHhi **(G)** and ITHlo **(H)** groups.

In this study, we quantitatively analyzed the CNV differences between the ITHhi and ITHlo groups. The results significantly revealed a higher proportion of aneuploidy in the ITHhi group (78%) compared to the ITHlo group (15%) (Figure 3A). Furthermore, the cell proportion analysis reported that the ITHhi group had less abundant OBs and macrophages, but a higher proportion of the NKT cells (Figure 3B). Then, the cell-type signature scores were evaluated in the TARGET dataset using the well-established marker gene sets. Compared to the subtype C2, the subtype C1 showed lower scores in B cells, T cells, and NKT cells, consistent with our prior GSEA and TME results (Figure 3C). Given that metabolic reprogramming plays a potential role in tumor heterogeneity, we analyzed the single-cell metabolism using the scMetabolism method. The results showed that the pyruvate metabolism, nicotinate and nicotinamide metabolism, glycolysis/gluconeogenesis, and N-glycan biosynthesis were all upregulated in the ITHhi group, whereas the pathways such as metabolism of xenobiotics by cytochrome P450, fatty acid elongation, and retinol metabolism were highly expressed in the ITHlo group (Figure 3D). TFs,which can bind to specific DNA sequences to regulate DNA transcription, are of vital importancein extensive biological processes. In our study, we utilized SCENIC software designed forsingle-cell transcriptomic data to assess the TF activity (Figure 3E). The results showed that theTFs including PHF20 (+), E2F1 (+), HOXB7 (+), ARID3A (+), TFDP1 (+), and XBP1(+) werehighly associated with the ITHhi group, while the TFs such as PLAGL1 (+), ATF3 (+), CREB5 (+), MAFF(+), HMGA1 (+), and EGR1 (+) showed higher activity in the ITHlo group. The mostrelevant TFs for each cell population were selected and visualized (Figures 3G,H). Furthermore,we assessed upregulation and downregulation of HALLMARK pathways between the subtypesvia the ssgsea function in the R package irGSEA. As a result, HALLMARK−GLYCOLYSIS, HALLMARK−MYC−TARGETS−V1, HALLMARK−MYC−TARGETS−V2, and HALLMARK−PI3K−AKT−MTOR−SIGNALING pathways were upregulated in the ITHhigroup, while these pathways in the ITHlo group were downregulated (Figure 3F).

### Remodeling of the cell communication network in the ITHhi and ITHlo subgroups

**FIGURE 4:**
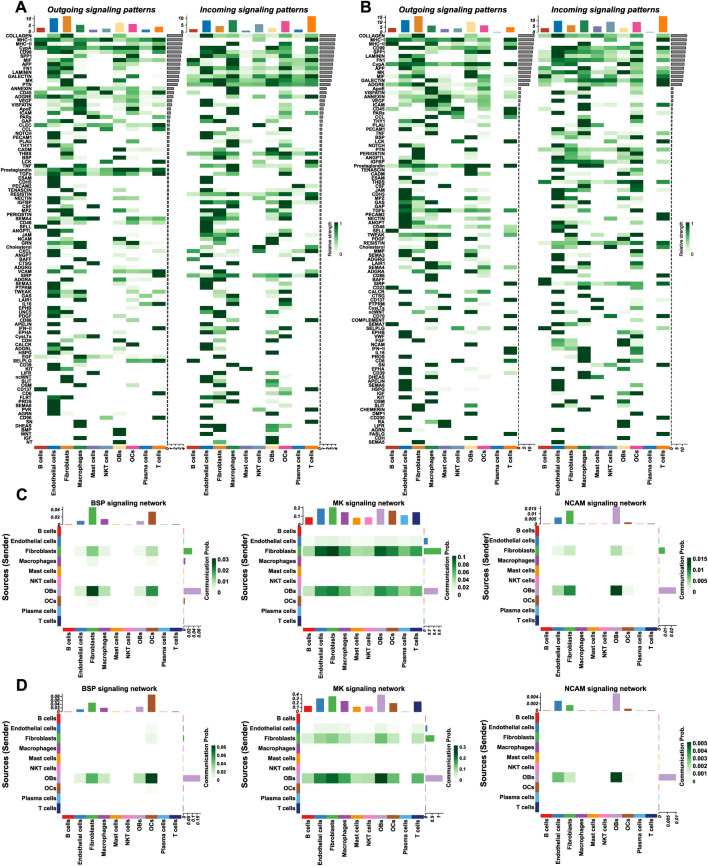
Intercellular communication patterns and signaling networks in ITH subtypes. **(A,B)** Heatmaps showing the comparative strength of outgoing and incoming signaling patterns among various cell types in the ITHhi **(A)** and ITHlo **(B)** groups. The color intensity indicates the relative strength of each signaling pathway. **(C,D)** Heatmaps illustrating the communication probability of selected signaling networks (BSP, MK, and NCAM) among different cell types in the ITHhi **(C)** and ITHlo **(D)** groups. The y-axis shows the sender cells, while the x-axis displays the receiver cells, with the color gradient representing the likelihood of communication.

The cell communication networks in the ITHhi and ITHlo samples were systematically compared using CellChat to investigate the intercellular interaction patterns between the high- and low-ITH states. Firstly, we evaluated the outgoing and incoming signaling patterns of 10 major cell populations. The contributions of each cell population as signal senders (outgoing signals) and receivers (incoming signals) were globally assessed to determine their impacts on the cellular communication networks under the different ITH states. Heatmap analysis visually revealed that each cell population uniquely contributed to the key signaling pathways, such as COLLAGEN, MIF, FN1, and LAMININ in the ITHhi group (Figure 4A). Conversely, it was suggested that fibroblasts and endothelial cells in the ITHlo group showed significant contributions to outgoing signals, serving as the main sources of extracellular matrix signals like collagen (COLLAGEN) and laminin (LAMININ). The signal receivers were widely distributed among the various cell populations (Figure 4B). Our results suggest that the high heterogeneity may reshape the roles of cells in intercellular communication patterns.

In this study, we extensively analyzed the changes in the 3 key signaling networks, including bone sialoprotein (BSP), midkine (MK), and neural cell adhesion molecule (NCAM). In the ITHhi group, the communication intensity of the BSP signaling network was comparatively lower, while the MK signaling communication was highly expressed, leading to changes in the action mode and intensity. Besides, the NCAM signaling network remained sparse (Figure 4C). Nevertheless, the regulation of the BSP, MK and NCAM pathways was significantly different in the ITHlo group. Specifically, the BSP signaling network showed a significant difference in the change of signal receivers. In the ITHhi group, the OBs predominantly transferred the BSP signals to the fibroblasts, whereas the OBs generated higher signaling to the OCs in the ITHlo group. The MK signaling remained highly active in the two groups, initiated by the fibroblasts and OBs, though its communication intensity was even higher in the ITHhi group. The NCAM signaling pathway remained sparse in both groups, but there were subtle changes in the interaction probability (Figure 4D). Our cell communication analysis results showed that the intercellular interaction networks were significantly different in the ITHhi and ITHlo states. Our findings potentially figured out the key signal sources and the target cells that drive the tumor microenvironment homeostasis and pathological progression, but also highlighted that the biological changes might be influenced by the ITH states through remodeling the cell-to-cell communication networks.

### Construction and validation of a prognostic model based on machine learning

**FIGURE 5:**
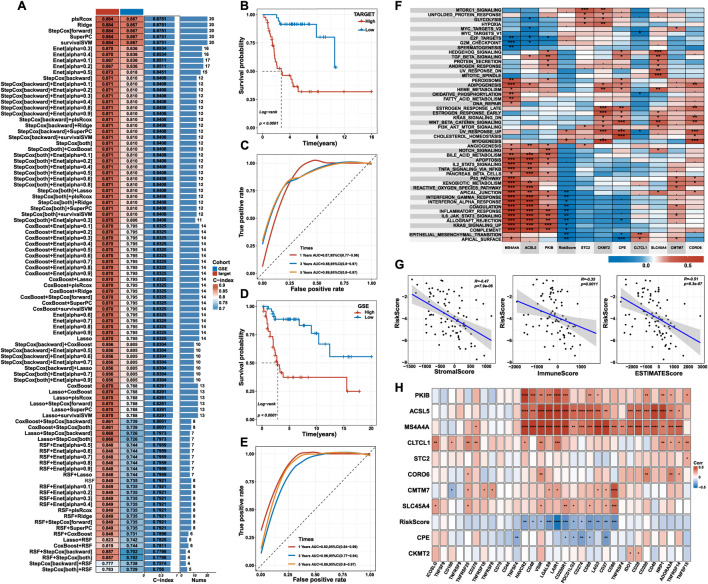
Construction and validation of a machine learning-based prognostic signature. **(A)** A comparison of C-indices among 94 machine learning algorithms to find the best prognostic model. **(B–E)** Kaplan-Meier survival plots **(B,D)** and time-dependent ROC plots **(C,E)** were utilized to evaluate the prognostic value of the signature in the TARGET **(B,C)** and GSE **(D,E)** cohorts. The log-rank test was employed to calculate the P-values for the survival analysis. **(F)** A heatmap illustrating the connection between model genes and biological pathways. **(G)** Scatter plots showing the relationship between the risk score and tumor microenvironment scores, such as Stromal, Immune, and ESTIMATE scores. **(H)** Examination of the relationship between the risk score, model genes, and immune checkpoint expression. *p < 0.05, **p < 0.01.

In our study, we selected 20 core genes that were expressed at different levels in the ITHhi and ITHlo groups and statistically associated with survival as a feature set to develop a reliable prognostic model. Then, we used the C-index to evaluate the performance quantitatively and applied machine learning algorithms and feature selection methods to our model. After screening the 94 method combinations, the StepCox [backward] + CoxBoost method with 10 selected core genes achieved the highest average C-index and was considered as the optimal model for the subsequent research in our study (Figure 5A). Using the risk scores derived from our model, all the patients in the TARGET dataset were stratified into the high-risk and low-risk groups. Based on the KM survival analysis, it was revealed that the low-risk patients showed significantly longer OS compared to the high-risk group (Log-rank *P* < 0.0001) (Figure 5B). Additionally, the time-dependent ROC curve analysis confirmed satisfactory predictive performance of our model, with AUC values of 0.87, 0.88, and 0.88 at 1, 3, and 5 years, respectively (Figure 5C). To assess its generalizability, our model was extensively validated in an independent external GSE dataset. The results demonstrated that our model can stratify the patients effectively, where the patients stratified by our model had a consistent prognosis (Log-rank *P* < 0.0001) (Figure 5D). In the GSE cohort, the AUC all reached 0.85 for the 1-, 3-, and 5-year survival, greatly supporting the high generalizability of our model (Figure 5E). To investigate the biological mechanisms underlying the risk scores, we assessed the correlation with the biological pathways based on the MSigDB database. Our results indicated that our risk score was highly associated with several pathways. Specifically, the risk score showed a significant negative correlation with the EPITHELIAL_MESENCHYMAL_TRANSITION, INTERFERON_GAMMA_RESPONSE, and COMPLEMENT pathways. Interestingly, the correlation patterns of certain model genes (e.g., MS4A4A and CKMT2) with these pathways were different from the risk score patterns, suggesting intricate regulatory relationships among the model genes (Figure 5F). Furthermore, the risk score showed a significant negative correlation with the stromal score (R = −0.47), immune score (R = −0.35), and ESTIMATE score (R = −0.51) (Figure 5G), potentially indicating that the high-risk state is associated with a “cold tumor” microenvironment with lower stromal and immune infiltration. Finally, we comprehensively assessed the correlations between the feature genes in our model and the immune checkpoint molecules. The heatmap analysis revealed that all the model genes except for the gene CPE were significantly positively related to the immune checkpoint genes (Figure 5H). Our reports may provide promising insights into the biological underpinnings of the prognostic values in our model.

### Protein-protein interaction analysis of model genes and drug analysis

**FIGURE 6:**
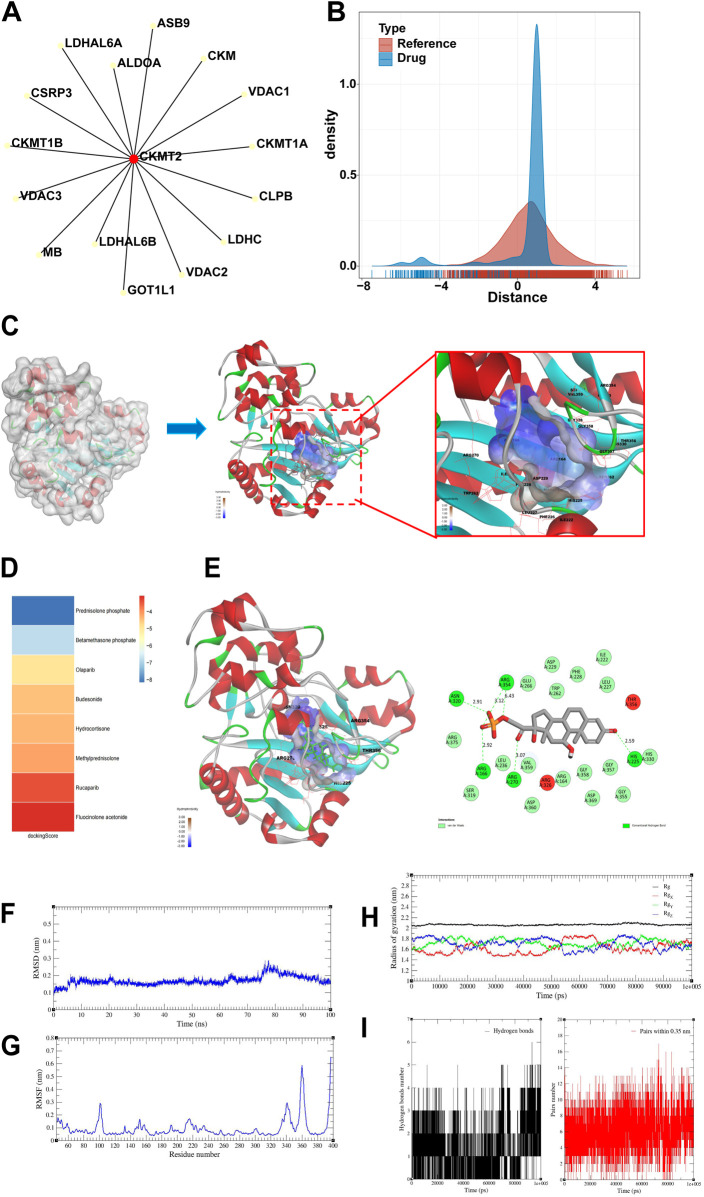
Interaction network, drug screening, and molecular dynamics simulation of CKMT2. **(A)** Protein-protein interaction (PPI) network visualizing the functional associations between CKMT2 and its interacting partners. **(B)** Density plot illustrating the distribution of perturbation distances for candidate drugs targeting the CKMT2-associated gene signature compared to reference controls. **(C)** Visualization of the 3D structure and active binding pocket of the CKMT2 protein. **(D)** Heatmap illustrating the molecular docking scores of candidate drugs with CKMT2. **(E)** 3D (left) and 2D (right) representations of the optimal binding mode between CKMT2 and Prednisolone phosphate. **(F–I)** Molecular dynamics simulation results of the CKMT2-Prednisolone phosphate complex over 100 ns: **(F)** Root Mean Square Deviation (RMSD) of the backbone atoms; **(G)** Root Mean Square Fluctuation (RMSF) of amino acid residues; **(H)** Radius of Gyration (Rg) indicating the compactness of the complex; and **(I)** Number of hydrogen bonds formed between the ligand and protein over time.

After multiple hypothesis testing using the random data derived from the reference, we screened 7 candidate drugs (OS.ITH.drug.txt) and selected the drug with small distances and FDR <0.001 as the candidate drug set associated with the CKMT2 gene set (Figure 6A). The distance-density histogram of the drugs related to the CKMT2 gene set is shown in Figure 6B. Statistical results are demonstrated in Figures 6C–I. During the 100 ns molecular dynamics simulation, the peak number of hydrogen bonds reached 6 in our study, and the average number of hydrogen bonds was 3 during the 0–70 ns period. After 80 ns, the average increased to 4, suggesting a change to a higher stability. The increase in hydrogen bond formation indicates that the prednisolone phosphate binds stably to the target CKMT2.

### Histochemical analysis and experimental validation of function

**FIGURE 7:**
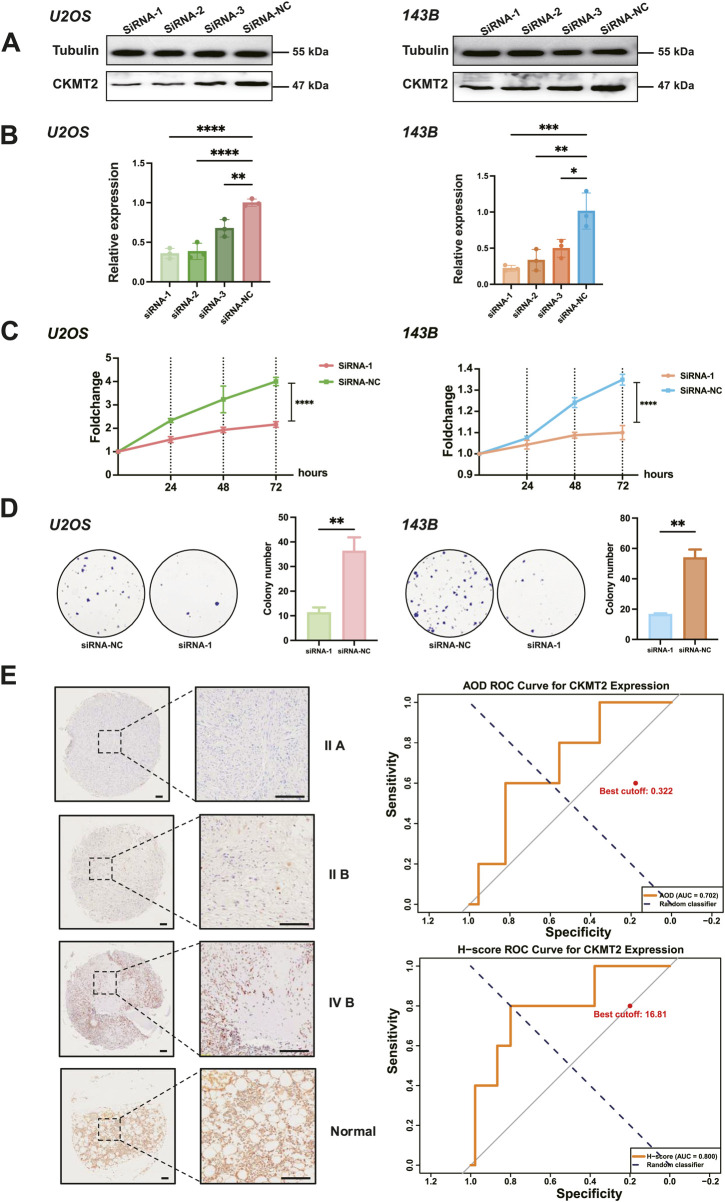
Experimental validation of CKMT2 expression and its oncogenic function in osteosarcoma. **(A,B)** Assessment of CKMT2 knockdown effectiveness in U2OS and 143B cell lines. Western blot **(A)** and RT-qPCR **(B)** analyses demonstrate a notable decrease in CKMT2 protein and mRNA levels in cells treated with siRNAs compared to the negative control (NC). *P < 0.05, **P < 0.01, ***P < 0.001, ****P < 0.0001. **(C)** Reducing CKMT2 expression hinders cell growth. CCK-8 tests demonstrate reduced viability of U2OS and 143B cells after CKMT2 is silenced at 24 and 48 h, with P < 0.0001. **(D)** Reduction in colony forming ability. Images and quantification from colony formation assays show reduced clonogenic potential in osteosarcoma cells lacking CKMT2. *P < 0.05, **P < 0.01, ***P < 0.001. **(E)** The expression of CKMT2 has clinical significance. Immunohistochemical staining shows increased levels of CKMT2 protein in osteosarcoma tissues (IIA, IIB, IVB) compared to normal tissues. ROC curve analyses (right) evaluate the diagnostic performance of CKMT2 expression.

In our study, we performed the CKMT2 knockdown experiments and validated the knockdown efficiency in the U2OS and 143B cell lines using the Western blot and qPCR analysis (Figures 7A,B). As shown in the CCK-8 assays and plate cloning experiments, the CKMT2 knockdown significantly declined the proliferation capacity of OS cells (Figures 7C,D). Additionally, there was higher CKMT2 expression in the high-grade OSs on staining tissue microarrays. Based on the AOD and h-score measurements, the diagnostic analysis of OS staging achieved promising results that the CKMT2 potentially is applicable as a diagnostic biomarker (Figure 7E).

## Discussion

Osteosarcoma is characterized by substantial clinical heterogeneity, leading to highly variable patient responses to multimodal treatment. While some patients achieve favorable outcomes, a significant proportion develop resistance, prompting the need to understand the underlying drivers of these disparate responses and explore strategies to re-sensitize resistant tumors. A fundamental contributor to this clinical challenge is intratumoral heterogeneity (ITH), as osteosarcoma tumors typically harbor a complex ecosystem of diverse cellular states. These states can reflect different levels of differentiation. They also relate to different outcomes (Bousquet et al., 2016; Espejo Valle-Inclan et al., 2025). Treatment can worsen this problem. After chemotherapy, radiotherapy, or targeted therapy, some aggressive cells can survive. Some cells may also gain new mutations. These changes can increase malignancy and support relapse. The fundamental driver of this extreme heterogeneity is profound genomic instability. Previous whole-exome and whole-transcriptome sequencing studies based on primary clinical osteosarcoma samples have revealed massive genomic rearrangements, such as chromothripsis, and the aberrant expression of repetitive DNA elements (Reimann et al., 2014; Ho et al., 2017a). These foundational genomic events continuously generate diverse cellular subclones, providing the raw material for the intratumoral heterogeneity we observe at the single-cell level.

In this work, we described heterogeneity from several angles. We started with single-cell transcriptomic data. We identified malignant cells using CNV inference. The detection of non-diploid signals within macrophage and osteoclast populations by CopyKAT warrants careful interpretation. Mature osteoclasts are inherently multinucleated, polyploid giant cells formed by macrophage fusion (Takegahara et al., 2016). Additionally, cell fusion events between tumor-associated macrophages and malignant cells are a recognized phenomenon in aggressive sarcomas, generating hybrid cells with aneuploid genomes (Pienta et al., 2022). Thus, these signals likely reflect true biological polyploidy and cell fusion dynamics rather than mere misclassification. We then quantified heterogeneity with ITH analysis. Next, we used stemness scoring. We also applied pseudotime analysis. These steps helped us infer cell-state transitions and possible trajectories. We paid special attention to high-ITH versus low-ITH tumor cells. We then asked what these groups “do” at the pathway level. We ran KEGG enrichment analyses in both groups. We also performed differential expression analysis using ITH-related genes, which enabled us to classify both the GSE and TARGET cohorts into two distinct molecular subtypes, designated C1 and C2. Importantly, the molecular signatures identified in this analysis align with the molecular fingerprints and differentially regulated networks previously discovered through whole-transcriptome analysis of fresh and FFPE osteosarcoma tissues (Ho et al., 2017b; Poudel and Koks, 2024), thereby providing independent validation of our findings. C1 tumors were characterized by prominent proliferative activity, as evidenced by enrichment of cell-cycle regulatory pathways, elevated spliceosome activity, and enhanced mitochondrial function; patients harboring C1 tumors exhibited significantly worse overall survival. In contrast, C2 tumors displayed a markedly distinct biological profile, with immune-related gene signatures being substantially more prominent.

We also compared metabolism across ITH groups. High-ITH tumors were enriched in glycolysis and pyruvate metabolism. Low-ITH tumors were linked to cytochrome P450 pathways. Fatty acid elongation signals were also higher in low-ITH tumors. We then explored regulation and interactions. We estimated transcription factor activity. We also analyzed cell–cell communication patterns. These results added functional context to the ITH-based groups.

We then aimed to translate these findings into a clinical tool. We wanted a risk model for prognosis prediction. We used ITH-related survival genes. We tested multiple machine-learning approaches. Some models achieved good C-index values. But many included too many genes. That limits clinical use. We therefore selected a more practical option. The combination of StepCox (backward) and CoxBoost gave a good balance. It kept gene count manageable. It also maintained strong performance. The final risk score was: RiskScore = −0.458×ACSL5 + 0.181×CKMT2 – 0.66×CLTCL1 – 0.709×CMTM7 + 0.531×CORO6 + 0.116×CPE – 0.077×MS4A4A – 0.355×PKIB – 0.575×SLC45A4 + 0.452×STC2. Using this score, we split the TARGET cohort into high-risk and low-risk groups for follow-up analyses.

We next examined pathway patterns linked to risk. Hallmark analysis showed EMT, KRAS signaling, and IL6–JAK–STAT3 pathways were negatively correlated with risk scores. This suggests these programs may be less active in high-risk cases. We also evaluated the immune microenvironment. StromalScore, ImmuneScore, and ESTIMATEScore were lower in the high-risk group. Immune infiltration was reduced. This points to a more immune-depleted tumor setting. It may help explain poorer outcomes in the high-risk group.

To improve translational relevance, we constructed a protein-protein interaction (PPI) network and performed drug-target docking. The utilization of established drug databases in this exploratory analysis is crucial, as it bridges our molecular discoveries with potential clinical applications, allowing us to identify existing drugs that could be repurposed to target specific vulnerabilities driven by intratumoral heterogeneity. Our molecular docking analysis revealed that CKMT2 exhibits stable predicted binding interactions with glucocorticoids, including prednisolone sodium phosphate. While this computational finding suggests a potential avenue for drug repurposing, it requires rigorous experimental validation. Mechanistically, glucocorticoids have been shown to modulate energy metabolism and exert anti-proliferative effects in osteosarcoma cells through glucocorticoid receptor (GR)-mediated transcriptional regulation, which may functionally converge with the role of CKMT2 in mitochondrial energy transfer. Furthermore, we also considered broader endocrine signaling. Although estrogen receptor (ER) biology in osteosarcoma remains debated—with some studies reporting ERα silencing by promoter hypermethylation and others suggesting its effects depend on p53 status (Wang et al., 2021)—targeting these metabolic and endocrine vulnerabilities may offer synergistic therapeutic opportunities. However, further *in vitro* and *in vivo* studies are necessary to confirm the efficacy and specific mechanisms of glucocorticoid-based or endocrine-targeted interventions in osteosarcoma.

We validated CKMT2 experimentally. While our initial prognostic model and drug screening were conducted *in silico*, we prioritized CKMT2 for subsequent *in vitro* functional validation because it emerged as the most critical candidate, exhibiting the highest variable importance in our machine learning models and occupying a central node in the PPI network. We knocked down CKMT2 using siRNA. WB and qPCR confirmed efficient suppression. CCK8 assays showed reduced proliferation. Colony formation assays showed reduced clonogenic ability. These results support an oncogenic role for CKMT2. We also examined clinical tissues. Immunohistochemistry on tissue microarrays showed CKMT2 overexpression is associated with poor prognosis. This supports its value as a biomarker.

The ROC AUC values for CKMT2 as a diagnostic biomarker (AOD: 0.702, H-score: 0.800) are moderate, suggesting that while CKMT2 expression shows promise as a diagnostic indicator, its utility as a standalone biomarker may be limited. Future studies should explore its diagnostic value in combination with other clinical and molecular markers.

Overall, our study provides a single-cell view of osteosarcoma heterogeneity. It links ITH status with prognosis and immune features. It also offers a clinically feasible risk model. CKMT2 emerges as a key gene with functional support. Future work should validate the model in larger multicenter cohorts, leveraging the growing body ofprimary clinical genomic data. It should also test combination therapies that target CKMT2 or related immunosuppressive programs, with the aim of improving response and survival in highly heterogeneous osteosarcoma.

## Conclusion

Our study utilized single-cell data and machine learning to build a risk model, which demonstrate that ITH is an important factor influencing the prognosis of osteosarcoma. Our findings provide novel multi-dimensional insights into the cellular and molecular mechanisms of osteosarcoma progression. We also identify the potential therapeutic target CKMT2. The interaction between CKMT2 and potential drugs was verified through molecular docking and kinetic simulations, and the role of CKMT2 in osteosarcoma cell lines was verified through molecular biology experiments. Overall, this study provides further insights into the development of new treatment methods for osteosarcoma.

## Data Availability

The original contributions presented in the study are included in the article/Supplementary Material, further inquiries can be directed to the corresponding authors.
