# Supplementary figures and images for: Heterogeneity-based stratification identifies CKMT2 as a prognostic marker in osteosarcoma
Source: Front Cell Dev Biol. 2026 May 25;14:1822741. doi: 10.3389/fcell.2026.1822741 (PMC13247562; doi:10.3389/fcell.2026.1822741)

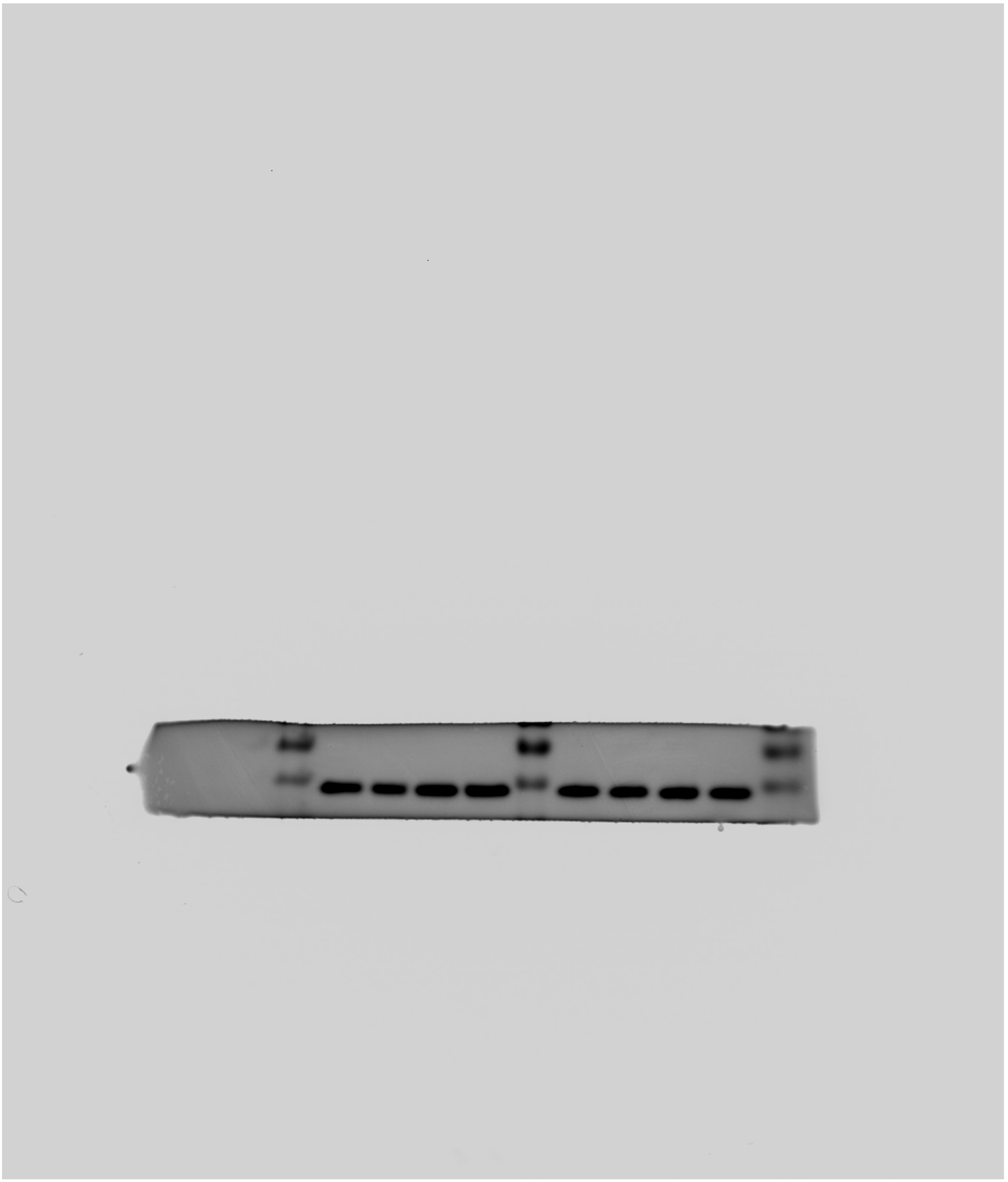

Supplement: Supplementary file 3 [file Image5.png]

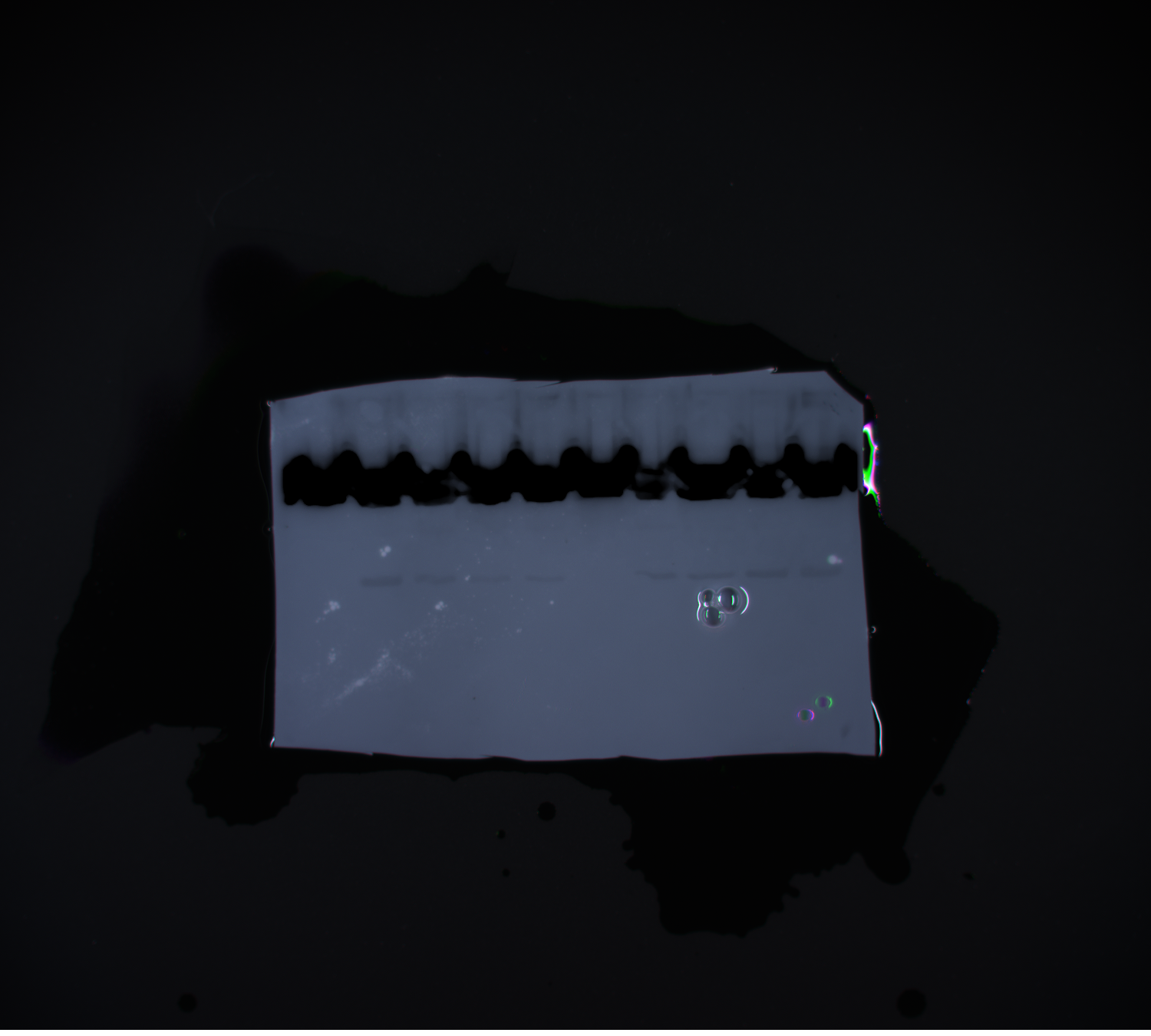

Supplement: Supplementary file 4 [file Image4.png]

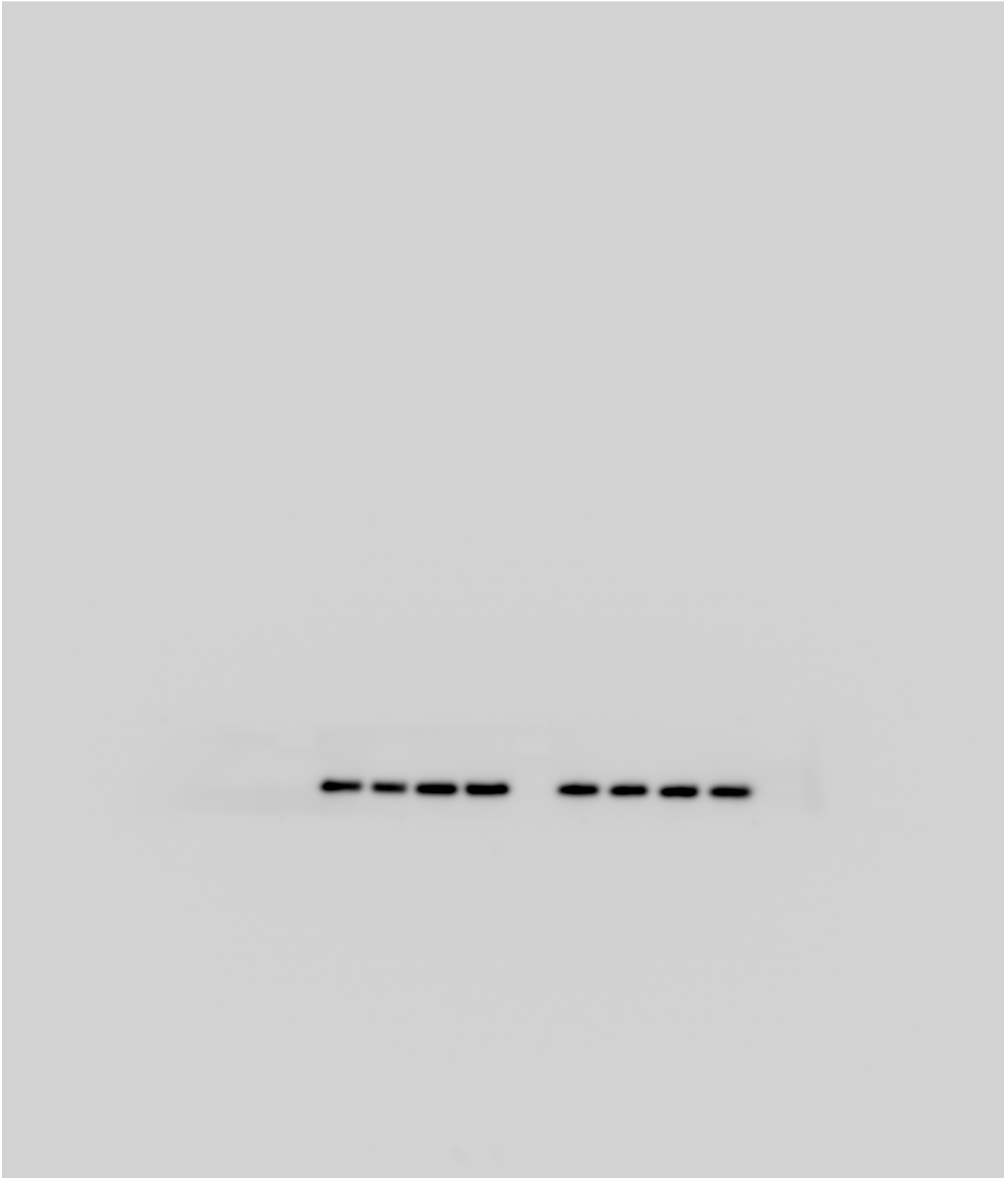

Supplement: Supplementary file 7 [file Image7.png]

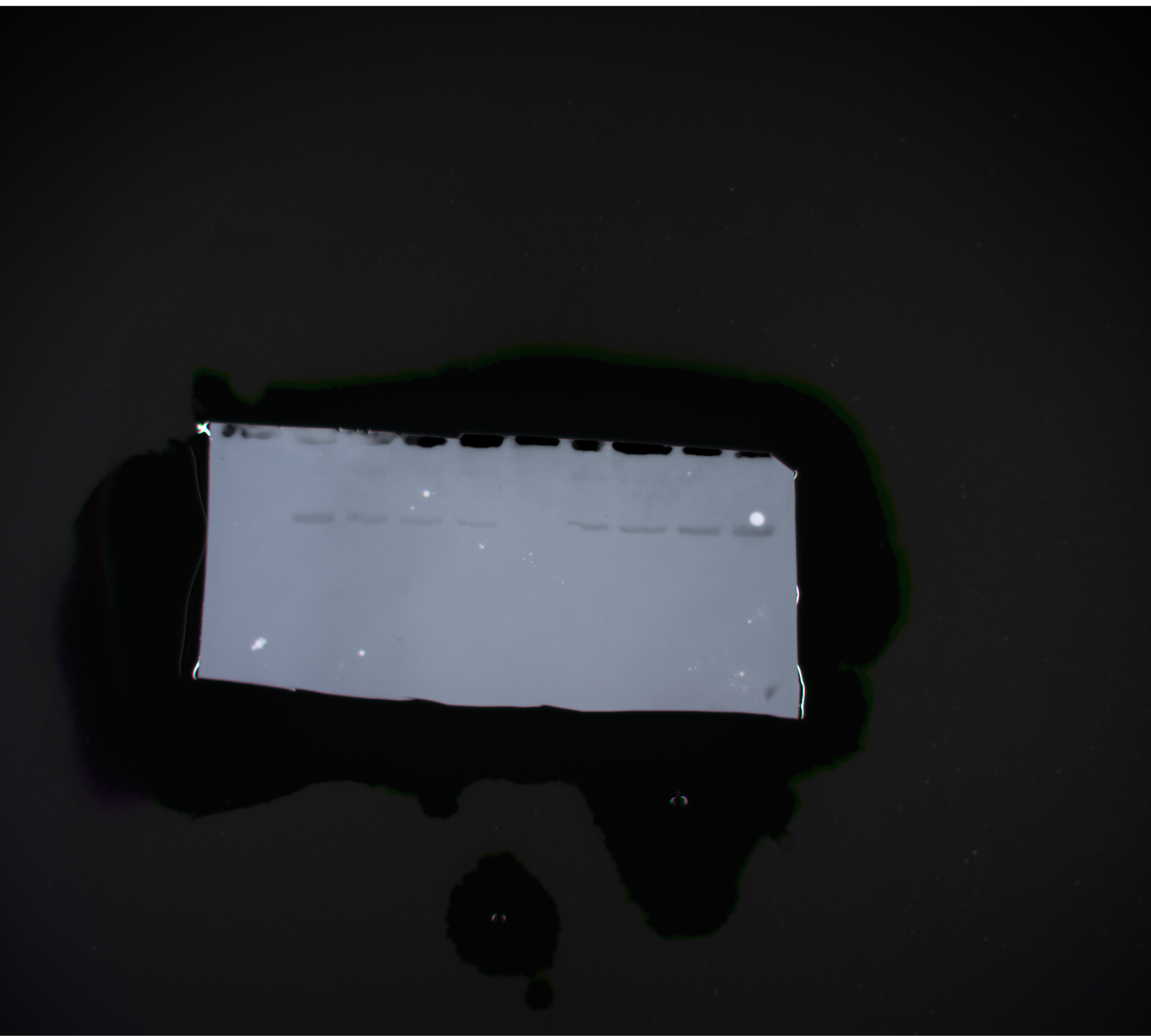

Supplement: Supplementary file 9 [file Image2.png]

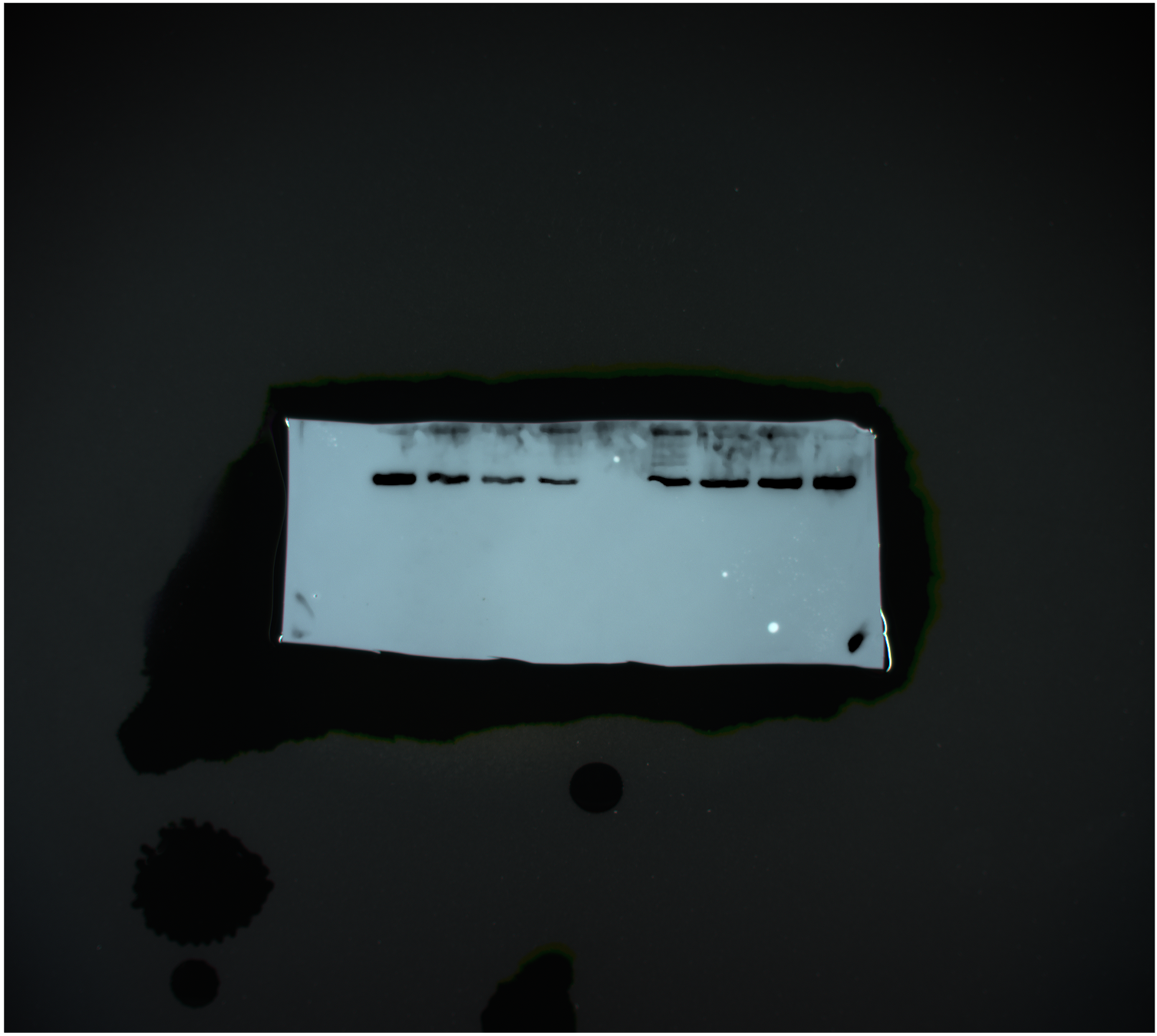

Supplement: Supplementary file 11 [file Image1.png]

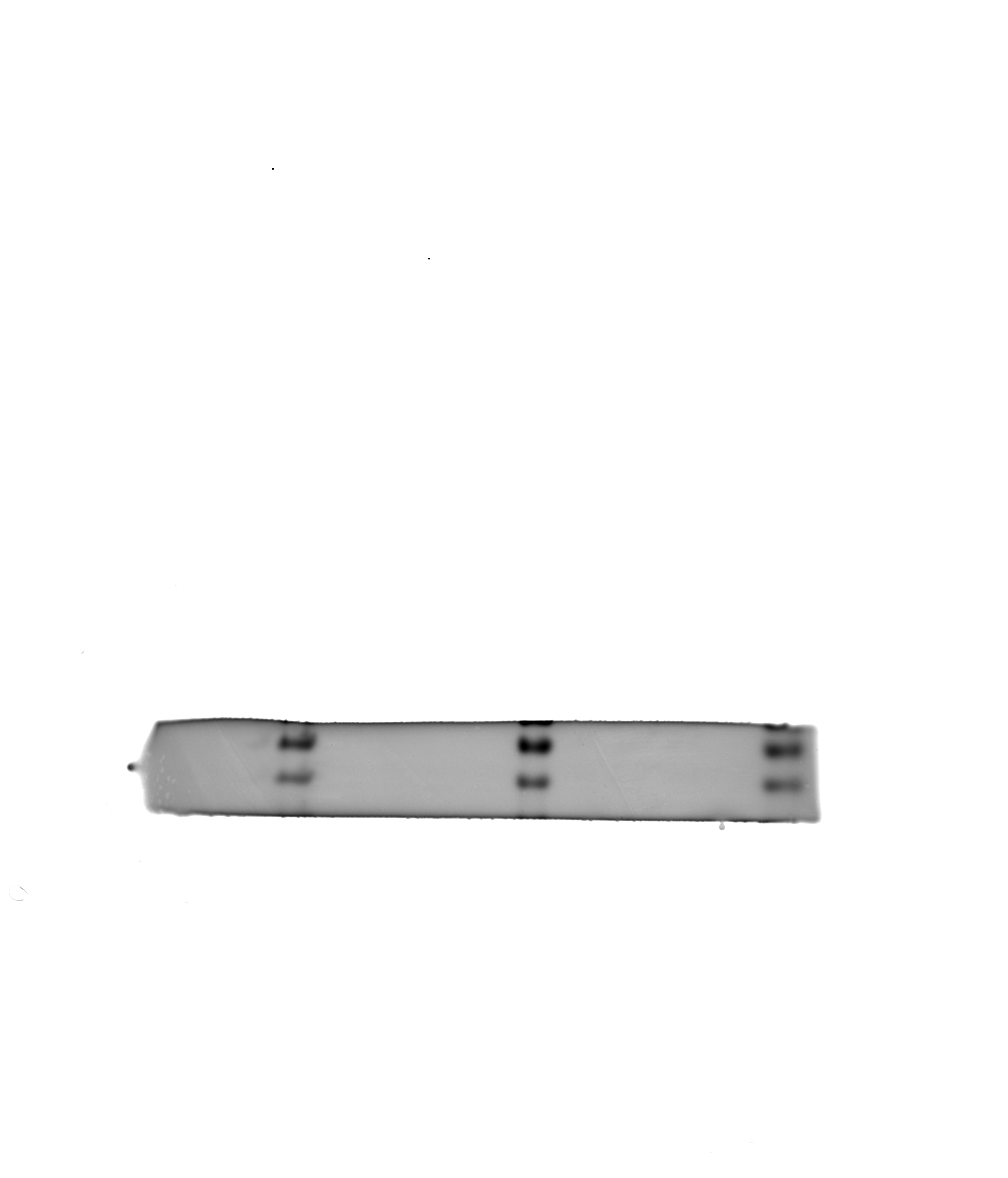

Supplement: Supplementary file 12 [file Image8.png]

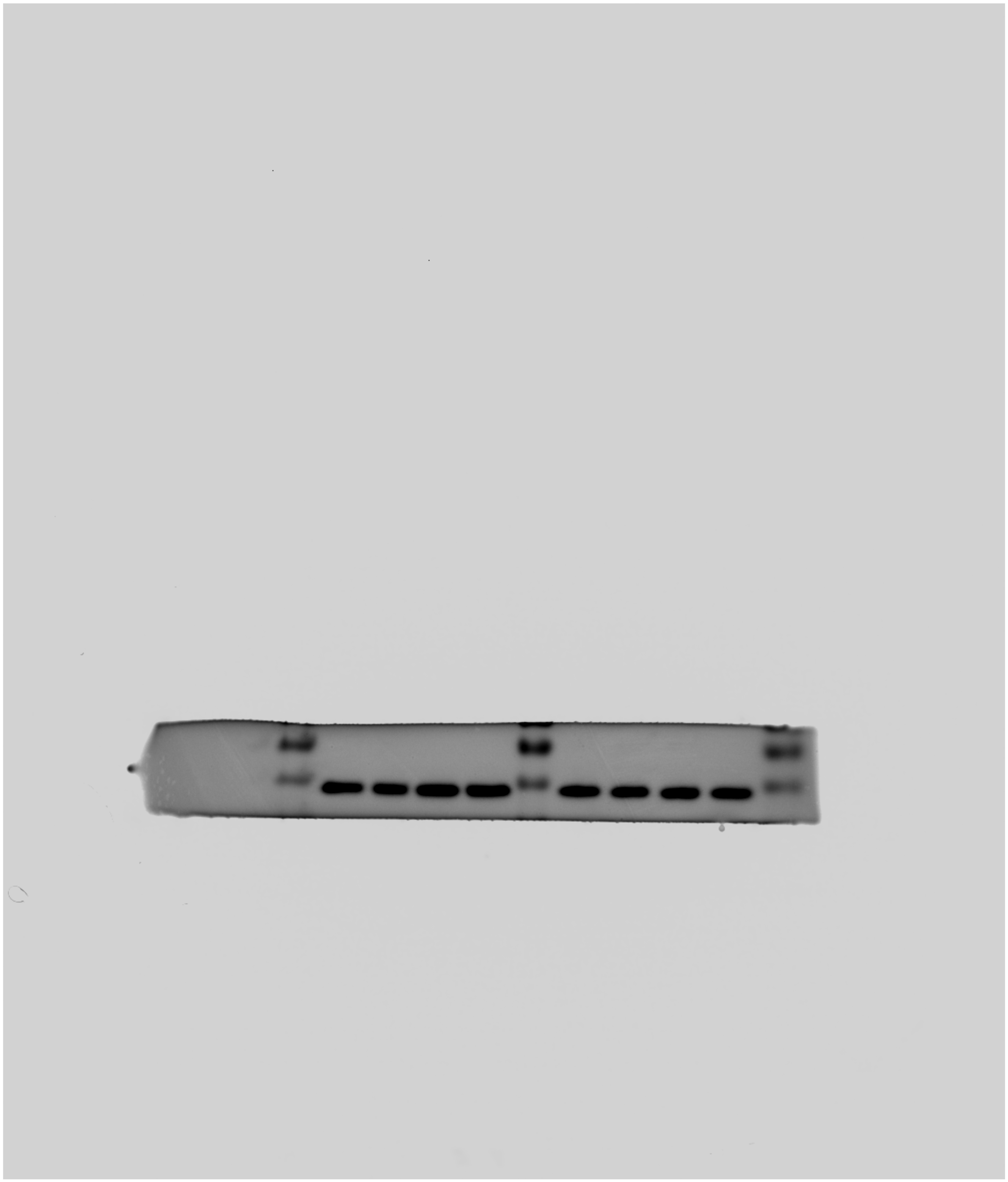

Supplement: Supplementary file 14 [file Image6.png]

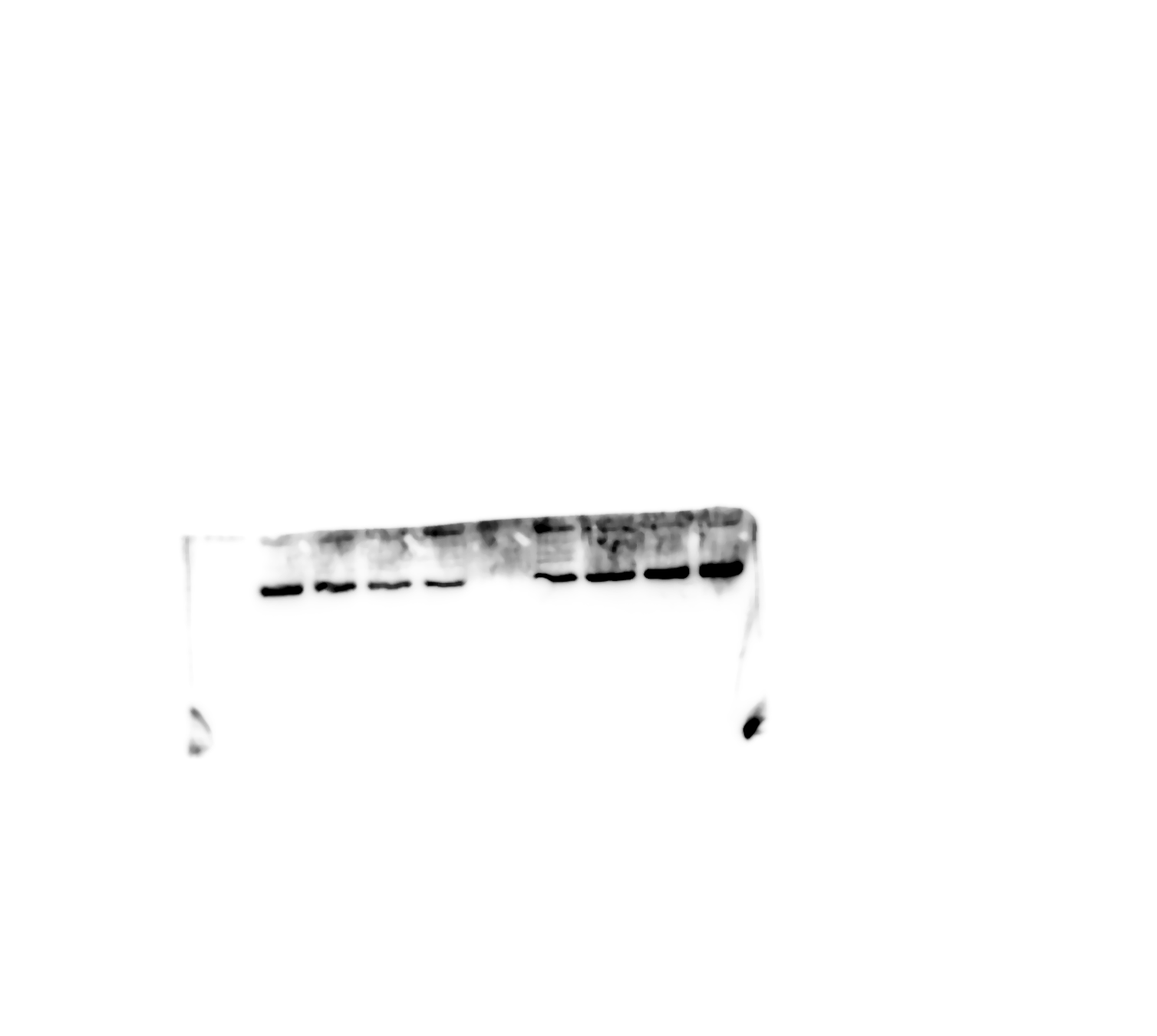

Supplement: Supplementary file 15 [file Image3.png]
